# Simultaneous versus video counting of coughs in hypertonic cough challenges

**DOI:** 10.1186/1745-9974-4-8

**Published:** 2008-09-09

**Authors:** Heikki O Koskela, Minna K Purokivi, Raija M Tukiainen

**Affiliations:** 1 Department of Respiratory Medicine, Kuopio University Hospital, P. O. Box 1777, 70211 Kuopio, Finland

## Abstract

**Background:**

The coughs occurring during cough provocation tests are usually counted at the same time when the test is being conducted, i.e., simultaneously. It is unknown whether cough counting from video recording might increase the accuracy of the cough counting. During recent years, cough challenges with hypertonic aerosols have been introduced. They often provoke very frequent coughing which may complicate the simultaneous cough counting.

**Objective:**

To assess whether cough counting from video recording is superior to simultaneous cough counting in two different hypertonic cough challenges.

**Methods:**

The analysis includes 82 hypertonic saline challenges performed on 66 subjects, providing 1984 observation minutes with both simultaneous and video cough counting. The cough sensitivity was expressed as the osmolality to provoke 15 cumulative coughs (CUM15). The analysis also includes 136 hypertonic histamine challenges performed on 114 subjects providing 5373 observation minutes with both simultaneous and video counting. The cough sensitivity was expressed as the cumulative number of coughs divided by the final histamine concentration administered (CCR). This challenge involved several additional measurements to cough counting.

**Results:**

For the saline challenge, the mean difference between the counting types was 0.0 coughs per minute with 95% limits of agreement of -1.2 to 1.2 coughs per minute. For the hypertonic histamine challenge the respective figures were 0.3 (-1.9 to 2.5) coughs per minute. At high coughing frequency the video counts tended to outnumber the simultaneous counts. The counting type had no effect on the hypertonic saline CUM15 and only a marginal effect on its repeatability. On the contrary, video counting resulted to significantly higher hypertonic histamine CCR values than simultaneous counting (p < 0.001).

**Conclusion:**

The agreement between simultaneous and video counting of coughs is generally good. However, as the coughing frequency increases, simultaneous counting may miss coughs, especially if the nurse has to share his/her attention to several activities simultaneously. Video recording is advisable for the hypertonic histamine challenge but unnecessary for the hypertonic saline challenge. To ensure reliable simultaneous cough counting, cough provocation tests should be performed in a quiet environment, applying as little unnecessary equipment and measurements as possible.

## Background

Cough provocation tests are mainly used for research purposes with capsaicin and citric acid being the most commonly used tussigens. The coughs are usually counted by a technician during the challenge and the test is stopped when a pre-determined number of coughs have been provoked. Usually a small number of coughs is required, from two to five coughs [1].

Recently we have evaluated hypertonic aerosols for cough provocation [2-6], mainly as a way to differentiate asthmatic cough from other types of cough. Our challenges differ from the traditional cough provocation tests in that the cough response has usually not been the end point of the challenge [2-4,6] and the subjects may cough vigorously, usually much more than the 2 – 5 coughs evoked during the traditional capsaicin and citric acid challenges. On the contrary to capsaicin and acid-provoked immediate cough response [7], hypertonic aerosol-provoked coughing usually appears after the nebulisation and can last several minutes [5]. Sputum induction may also occur. To ensure that all of the coughs are recorded we have videotaped the challenges in our last two studies [5,6]. Cough counting from video recording has been regarded as the 'gold standard' since it allows visualisation of the subjects' movements as well as the audibility of the characteristic sound to verify coughs. It also offers the possibility to view the recording repeatedly in any cases of uncertainty [8-10]. In our studies, trained nurses have counted the coughs during the challenge, and after the study has been completed, the coughs have also been counted from the video recordings. In the publications, we have only utilised the cough counts from the video recordings [5,6].

Video recording makes the hypertonic challenges more complicated and laborious and may thus hinder the widespread adoption of these challenges. In the present study we hypothesised that video recording is not essential. Therefore, we have compared the numbers of hypertonicity-provoked coughs that have been counted during the challenge with those counted afterwards, from video recordings [see additional file 1].

## Methods

### Subjects

The present analysis is based on two adult patient populations which were recruited for two clinical studies investigating hypertonicity-provoked cough. The study utilising a saline challenge [5] included nineteen healthy subjects, 26 asthmatic patients with chronic cough, and 21 non-asthmatic patients with chronic cough. There were 23 men and 43 women, with a mean (SD) age of 50 (12) years. Eighteen patients repeated the saline challenge in order to evaluate the repeatability of the responses.

The study utilising hypertonic histamine challenge [6] included 25 healthy subjects, 30 asthmatic patients, and 82 non-asthmatic patients with respiratory symptoms. There were 57 men and 80 women, mean 46 (12) years. The Finnish National Agency of Medicines and the Institutional Ethics Committee approved the studies and all subjects provided their informed written consent.

### The saline challenge

A detailed description of the challenge has been published previously [5]. Fifteen minutes prior to the challenge, the subjects inhaled four 0.1 mg puffs of salbutamol to prevent bronchoconstriction. Spirometry was measured before and after salbutamol, as well as after the final saline concentration. The challenge consisted of serial two-minute inhalations of phosphate-buffered saline using a high-output ultrasonic nebuliser (DeVilbiss Ultraneb 3000, Sunrise Medical Ltd, West Midlands, UK). By adjusting the saline concentration, a stepwise increase in the osmolalities of the solutions was achieved: 300, 600, 900, 1200, 1500, 1800 and 2100 mOsm/kg. The coughs were counted during each two-minute inhalation as well as for two minutes after the inhalation. The response was expressed as the osmolality to provoke 15 cumulative coughs (CUM15).

### The hypertonic histamine challenge

A detailed description of the challenge has been published previously [11]. Spirometry was performed before the challenge. The challenge consisted of serial two-minute inhalations of histamine diphosphate dissolved in hypertonic phosphate-buffered saline using a low-output ultrasound nebuliser (Omron U1; Omron LTD; Tokyo, Japan). The histamine concentrations of the solutions were 0.0075, 0.015, 0.03, 0.06, 0.125, 0.25, 0.5, 1.0, 2.0, 4.0, and 8.0 mg/ml. The osmolality of the solutions remained as constant (1522 – 1577 mOsm/kg). The coughs were counted during each two-minute inhalation as well as for one and a half minutes after the inhalation. At that stage, spirometry was again performed, after every histamine concentration. The challenge was terminated when a 20% fall in forced expiratory volume in one second was demonstrated. The cough response was expressed as the cumulative number of coughs divided by the final histamine concentration administered (CCR) [4].

### Cough counting

Before the studies, the coughs were defined as a forced expulsive manoeuvre, usually against a closed glottis and which was associated with a characteristic sound [12]. Special emphasis was paid to ensure the exclusion of sounds caused by throat clearing etc. All challenges were video recorded. Coughs were counted by two trained study nurses during the challenge (the 'simultaneous counting'). After the studies, the coughs were also counted from the video recordings either by the more experienced study nurse or by one of the authors (the 'video counting').

### Data analysis and statistics

The coughs occurring during the saline challenge were counted in one-minute periods. The numbers of simultaneously and video counted coughs were compared with each other. The coughs occurring during the hypertonic histamine challenge were counted in a single two-minute period during the inhalation and in a single 1.5-minute period after the inhalation. The coughs occurring during these periods were expressed as coughs per minute and again, the numbers of simultaneously and video counted coughs were compared with each other. To express agreement of the counting methods, Bland-Altman plots [13] were used and 95% limits of agreement were determined. Intraclass correlation coefficient was used to express repeatability [14]. In addition, linear regression analysis and Student's t-test were utilised when appropriate. Log-transformed data of hypertonic histamine CCR were applied as these values were log-normally distributed. Means and 95% confidence limits are expressed if not stated otherwise.

## Results

Two saline challenges lacked video recordings due to technical problems with the video recorder. The analysis thus includes 82 saline challenges performed on 66 subjects, providing 1984 observation minutes with both simultaneous and video cough counts. Ten hypertonic histamine challenges lacked video recordings due to similar technical reasons. In addition, twelve hypertonic histamine challenges lacked simultaneous cough counts mainly due to technical problems with the nebuliser that completely drew the nurse's attention. In one subject neither simultaneous nor video cough counts were available. The analysis thus includes 136 hypertonic histamine challenges performed on 114 subjects providing 5373 observation minutes with both simultaneous and video counts.

**Figure 1:**
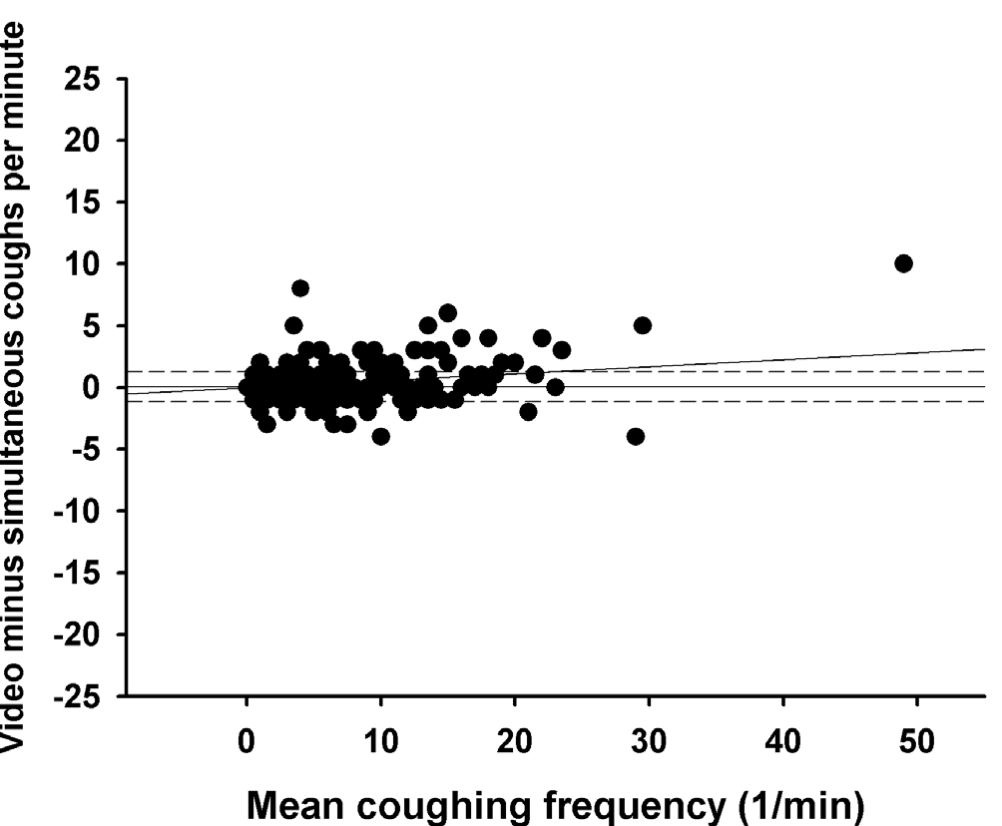
**The Bland-Altman plot for cough counts in hypertonic saline challenge. **The difference between video vs. simultaneously counted coughs for each observation minute is plotted against the mean of the counted coughs per minute. The solid horizontal line represents the mean difference between the two counting methods and the dashed lines the 95% limits of agreement. The oblique line indicates the regression line.

**Figure 2:**
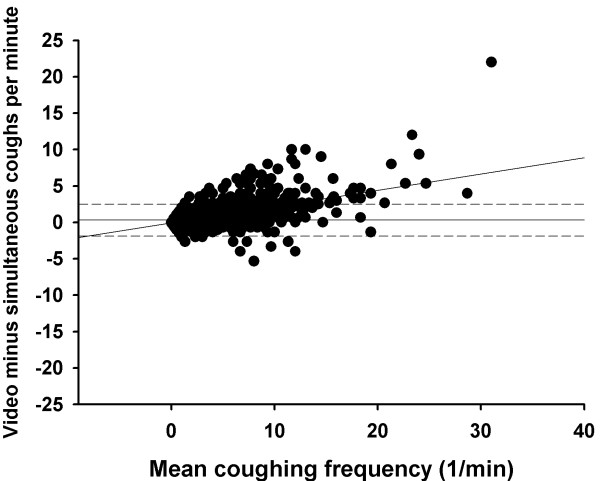
**The Bland-Altman plot for cough counts in hypertonic histamine challenge.** The difference between video vs. simultaneously counted coughs for each observation minute is plotted against the mean of the counted coughs per minute. The solid horizontal line represents the mean difference between the two counting methods and the dashed lines the 95% limits of agreement. The oblique line indicates the regression line.

During the entire saline challenge, simultaneous counting detected mean 16.8 (11.6 – 21.9) coughs whereas video counting detected 17.2 (11.9 – 22.5) coughs (p = 0.23). During the entire hypertonic histamine challenge, simultaneous counting detected mean 52.3 (43.6 – 61.0) coughs and the video counting 65.0 (53.9 – 76.1) coughs (p < 0.001). The Bland-Altman plots of the video and simultaneously counted coughs are shown in figures 1 and 2. The mean difference between video and simultaneously counted coughs in the saline challenge was 0.0 coughs per minute, with 95% limits of agreement of -1.2 to 1.2 coughs per minute. For the hypertonic histamine challenge the mean difference was 0.3 coughs per minute and the 95% limits of agreement were -1.9 to 2.5 coughs per minute.

The Bland-Altman plots also show that the video counted coughs tended to outnumber those counted simultaneously when the coughing frequency increased. This can be shown utilising linear regression analysis with the difference in the counted coughs as the dependent variable and the mean coughing frequency as the independent variable: R = 0.31, p < 0.001 for the saline challenge and R = 0.63, p < 0.001 for the hypertonic histamine challenge (figures 1 and 2).

**Figure 3:**
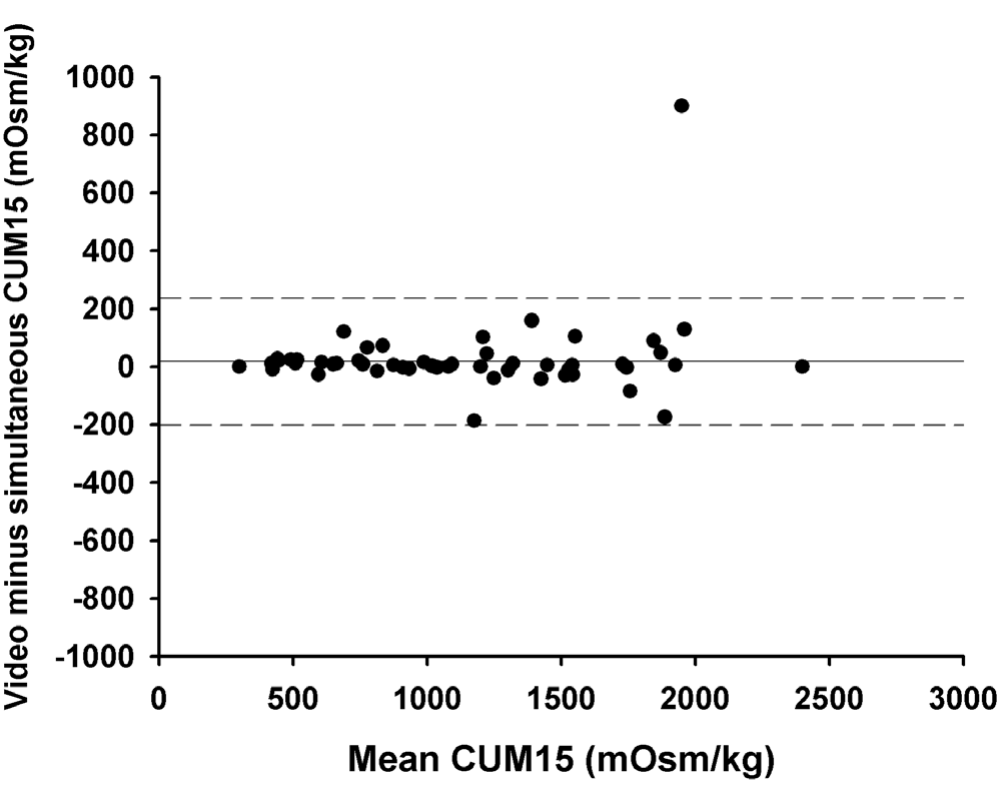
**The Bland-Altman plot for CUM15, the index that was used to express cough sensitivity in hypertonic saline challenge.** The difference between video vs. simultaneously counted CUM15 is plotted against the mean of the respective index. The solid horizontal line represents the mean difference between the two counting methods and the dashed lines the 95% limits of agreement.

**Figure 4:**
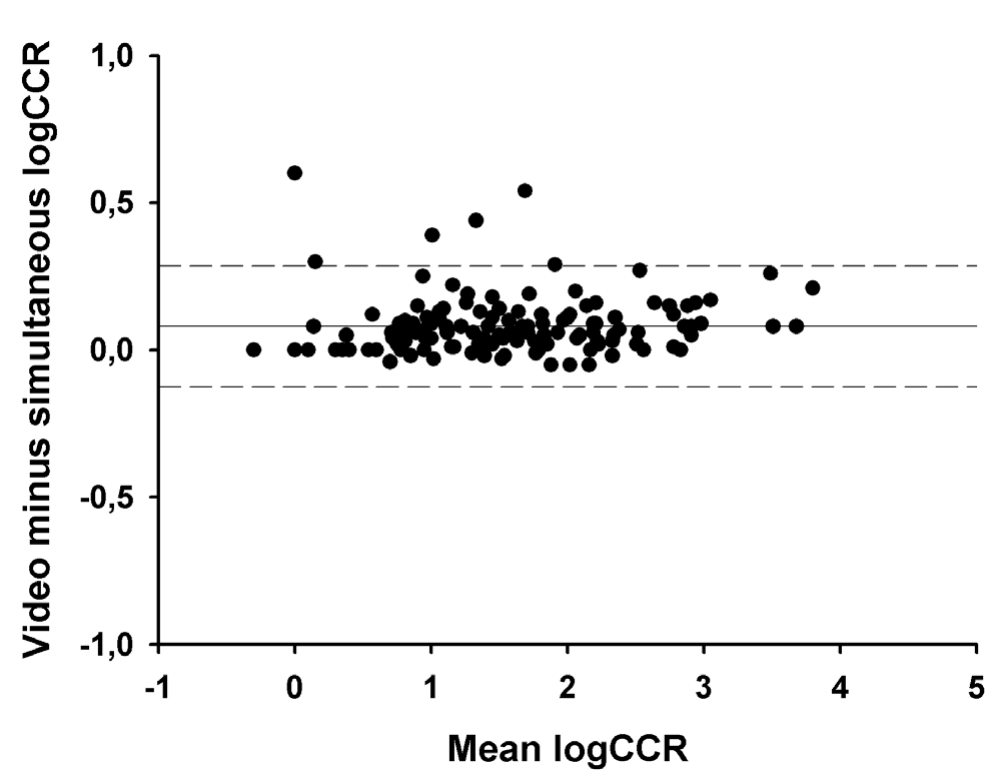
**The Bland-Altman plot for CCR, the index that was used to express cough sensitivity in hypertonic histamine challenge. **The difference between video vs. simultaneously counted, log-transformed CCR is plotted against the mean of the respective index. The solid horizontal line represents the mean difference between the two counting methods and the dashed lines the 95% limits of agreement.

For the saline challenge, the mean CUM15 was 1775 (1602 – 1947) mOsm/kg when utilising simultaneous counts and 1788 (1615 – 1961) mOsm/kg when utilising video counts (p = 0.37). For the hypertonic histamine CCR the respective values were (geometric means and 95% confidence intervals) 32.8 (22.6 – 47.8) and 40.2 (27.4 – 58.8) coughs per mg/ml (p < 0.001). The Bland-Altman plots for CUM15 and CCR values are presented in figures 3 and 4.

As eighteen patients underwent two identical saline challenges within 2 – 14 days, it was possible to analyse the repeatability of the saline CUM15 using both simultaneously and video counted coughs. The respective ICC values were 0.81 and 0.90 reflecting slightly better repeatability of the saline challenge response when video counts were utilised.

## Discussion

The present study shows that the agreement between simultaneous and video counting of coughs during hypertonic challenges is generally good. However, as the coughing frequency increases the video counted coughs may outnumber those counted simultaneously. This finding suggests that when a subject coughs frequently, the study nurse may have difficulties in catching all the coughs when she/he is simultaneously conducting the challenge.

According to our trained nurses there may be several reasons why some coughs were missed during the simultaneous counting. First, during the challenge the nurse has to concentrate on several activities in addition to cough counting. These include video recording, monitoring the function of the nebuliser, filling and emptying the container of the nebuliser, using the spirometer, as well as caring for the study subject. Second, there may be several types of interruptions during the challenge, including sounds outside the room, possible visitors, and phone calls. On the contrary, during the viewing of a video recording the nurse can completely concentrate on the counting. In case of interruptions or uncertainty about the nature of a breath sound the recording can be re-wound and viewed and heard as many times as needed. Third, the nurses felt that the sound recording of the video camera highlights the sounds generated by the study subject while the background sounds arising elsewhere are muted. These comments suggest that in order to ensure reliable simultaneous cough counting and patient safety, cough provocation tests should be performed in a quiet environment without interruptions, applying as little unnecessary equipment and measurements as possible.

These issues may explain the observation that the differences in video vs. simultaneous cough counts were greater during the hypertonic histamine challenge than during the saline challenge. The nebuliser used in the former challenge functioned less reliably and the nurses often had to service it during the challenge. In addition, the hypertonic histamine challenge included a spirometric evaluation after every histamine concentration whereas spirometry was performed only at the beginning and at the end of the saline challenge.

The type of cough counting had no effect on CUM15, the index that was used to express the cough responsiveness to the hypertonic saline challenge, and only a marginal effect on the repeatability of this challenge. Therefore, video recording of the hypertonic saline challenge seems to be unnecessary. This is probably true for traditional cough provocations with capsaicin and citric acid as well. They usually end when five coughs have been provoked and such a low frequency coughing can be reliably counted simultaneously.

In contrast, video recording is advisable during the hypertonic histamine challenge. The type of cough counting had a statistically significant effect on CCR with video counting showing larger values than simultaneous counting. This was due to the fact that simultaneous counting often missed coughs at high coughing frequencies probably because the nurse had to share her attention to several activities simultaneously. The authors believe that video recording is also useful in other types of cough provocation tests that include several measurements and devices used simultaneously.

In the present study the individual counting the coughs from the video recording was not blinded from the results of the simultaneous counts, which may be regarded as a weakness of the study. In fact, both simultaneous and video cough counts were usually performed by the same, highly experienced study nurse (RT). We feel that this is not simply a weakness, as by this means the criteria for coughs remained the same, with the type of counting (video vs. simultaneous) being the only factor that varied.

## Conclusion

Though the agreement between simultaneous and video counting of coughs during hypertonic challenges is good, simultaneous counting may miss coughs occurring at high frequencies. Utilisation of video recording to count coughs had no effect on the hypertonic saline challenge end point but significantly affected the hypertonic histamine challenge end point. Video recording is therefore advisable for the latter but not for the former challenge. To ensure reliable simultaneous cough counting and patient safety, cough provocation tests should be performed in a quiet environment without interruptions, applying as little unnecessary equipment and measurements as possible.

## Competing interests

The authors declare that they have no competing interests.

## Authors' contributions

HK and MP planned the studies. HK, MP and RT recruited the subjects. RT performed most of the challenges and counted the coughs. HK analysed the results and wrote the manuscript. All authors read and approved the final manuscript.

## Supplementary Material

Additional file 1 Simultaneous versus video counting of coughs in hypertonic cough challenges. The data provided represent the statistical analysis of the difference between simultaneous and video counting of coughs in two hypertonic cough challenges. Click here for file
