# Supplementary material for: Simultaneous versus video counting of coughs in hypertonic cough challenges
Source: Cough. 2008 Sep 9;4:8. doi: 10.1186/1745-9974-4-8 (PMC2542393; doi:10.1186/1745-9974-4-8)
Supplement: Additional file 1 — Simultaneous versus video counting of coughs in hypertonic cough challenges. The data provided represent the statistical analysis of the difference between simultaneous and video counting of coughs in two hypertonic cough challenges. [file 1745-9974-4-8-S1.doc]

**UTILITY OF COUGH RESPONSE DURING HYPERTONIC HISTAMINE CHALLENGE IN DIAGNOSING ASTHMA**

**Corresponding author:**

Minna Purokivi, MD, PhD

Kuopio University Hospital

P.O.Box 1777

70211 Kuopio

Finland

e-mail: [minna.purokivi@kuh.fi](mailto:minna.purokivi@kuh.fi)

telephone: +358 447174795

Fax: +358 17 172683

Heikki O. Koskela, MD, PhD, Department of Respiratory Medicine, Kuopio University Hospital, P.O.Box 1777, 70211 Kuopio, Finland e-mail: heikki.koskela@kuh.fi

Tiina Koistinen, MD, Department of Otorhinolaryngology, Kuopio University Hospital, P.O.Box 1777, 70211 Kuopio, Finland e-mail: tiina.koistinen@kuh.fi

Jarkko Magga, MD, PhD, Department of Internal Medicine, Kuopio University Hospital, P.O.Box 1777, 70211 Kuopio, Finland e-mail: jarkko.magga@kuh.fi

Keijo Peuhkurinen, MD, PhD, Department of Internal Medicine, Kuopio University Hospital, P.O.Box 1777, 70211 Kuopio, Finland e-mail: keijo.peuhkurinen@kuh.fi

Vesa Kiviniemi, PhLic, IT Centre, Statistical and Mathematical Services, University of Kuopio, P.O. Box 1627, 70211 Kuopio, Finland e-mail: vesa.kiviniemi@uku.fi

Kirsi M. Kontra, Pharm.Lic., Department of Pharmacy, Kuopio University Hospital, P.O.Box 1777, 70211 Kuopio, Finland e-mail: kirsi.kontra@kuh.fi

Running title: HHC Induced Cough in Diagnosing Asthma

ABSTRACT

Airway responses to bronchial provocation tests are traditionally assessed with spirometry which necessitates considerable patient co-operation. It has been shown that coughing during bronchial provocation tests is related to the degree of bronchoconstriction which, in turn, is independent of patient co-operation. The aim of the present study was to evaluate the utility of coughing induced by the hypertonic histamine challenge in the differential diagnosis of asthma in a clinically relevant patient population.

The study population consisted of 25 healthy volunteers, 30 asthmatics, and 82 non-asthmatic subjects with respiratory symptoms due to other diseases. Hypertonic histamine solution was administered with ultrasonic nebuliser with the challenges being videotaped. The cough response was expressed as the cumulative number of coughs divided by the final histamine concentration administered (CCR).

The geometric mean (95% CI) of CCR for asthmatics was 302 (166-562) coughs per mg/ml, for the symptomatic controls 29.5(20.0-43.7) coughs per mg/ml (p<0.001) and for the healthy controls 6.61(3.02-14.5) (p<0.001) coughs per mg/ml. According to the ROC curve, the optimal cut-off point for logCCR was 2.22, with the specificity and sensitivity to detect asthma among symptomatic subjects being 86% and 70%.

Cough response to hyperosmolar challenge may be useful in the differential diagnosis of airway diseases.

KEYWORDS

asthma, cough, histamine, sensitivity and specificity

**INTRODUCTION**

Traditionally the diagnosis of asthma has been based on the measurement of airway hyperresponsiveness and reversible bronchoconstriction [1] but these are known to require considerable patient co-operation especially in spirometry. Nowadays, patients are often elderly, and those who cannot perform spirometry are not uncommon.[2] [3] Therefore, complementary methods are needed to replace the FEV_1_ measurement. Both pulse oximetry and observation of wheezing with chest auscultation have been utilized as adjunct techniques, or instead of, spirometry in young children [4-7] and in adults [8] while assessing objectively the responses during inhalation challenges.

One previously recognised sign of bronchoconstriction is the cough induced by the different inhalation challenges. Since this cough is independent of compliance on the part of the patient, it could be used as a surrogate for spirometry. We have already shown that coughing frequency during airway challenge with histamine is closely related to the degree of induced bronchoconstriction.[9] In addition, the indirect inhalation challenges, such as mannitol and hypertonic saline solution challenge, induce frequent coughing which already has clinical implications. [10] However, this cough, is not solely dependent on the presence of bronchoconstriction.

One problem encountered in the diagnosis of asthma is its differentiation from several pulmonary and cardiac disorders whose probability increases in parallel with aging, for example, overt heart failure [11], COPD [12], sarcoidosis [13], gastroesophageal reflux and postnasal drip syndrome [14]. Therefore, the diagnostic value of a hyperresponsiveness test cannot be reliably determined with a study population consisting only of young, steroid-naïve asthmatics and healthy, non-symptomatic subjects. [9] In order to create a scenario corresponding to an unselected out-patient clinic material, both asthmatic subjects and subjects with other respiratory co-morbidities common in the adult population were recruited.[15] The aim of this study was to evaluate the utility of hypertonic histamine challenge (HHC) induced cough in the differential diagnosis of difficult-to-diagnose asthma in a real-life clinical setting.

**MATERIALS AND METHODS**

A detailed description of the study population and the challenge has been published previously, and this is another analysis of the same data set. [15] The previous report concerned the role of HHC induced airway hyperresponsiveness in the differential diagnosis of asthma in a clinically representative, symptomatic group of subjects. The present report describes the role of cough responses in the same patient population.

**Subjects**

A total of 138 subjects took part in this study. According to the GINA guidelines [16], a total of 31 of these subsequently recruited subjects were confirmed as having asthma. One of them had intermittent, 26 had mild persistent and 4 suffered from moderate persistent asthma (viite 14). The cough recording succeeded in 30 subjects. The symptomatic control group consisted of the following subjects: 26 subjects with symptoms suggestive of asthma but without objective evidence of reversible airway obstruction in at least 6 months follow-up, seven subjects with COPD [17], 15 patients with parenchymal lung disease [18], 21 subjects with persistent rhinitis [19], and 13 subjects with chronic NYHA I-II chronic heart failure [20]. In addition, 25 healthy, non-symptomatic subjects were recruited from the personnel of Kuopio University Hospital. Further details of the groups are described in table 1.

The exclusion criteria for all subjects were FEV_1_ < 65%, severe coronary heart disease, and severe heart failure or arrhythmia tendency. The use of oral or inhaled corticosteroid preparations was not allowed during the four weeks prior to the study. The dose of oral steroids in subjects with parenchymal lung diseases had to be less than 10 mg per day. Before the inhalation challenge test, the patients refrained from taking short-acting β2-agonists for six hours, long acting β2-agonists and tiotropium for 48 hours, anticholinergics for 8 hours, antihistamines and leukotriene receptor antagonists for 3 days. In addition, the subjects were asked to avoid smoking, coffee and vigorous exercise during the study day. The Finnish National Agency of Medicines and the Institutional Ethics Committee approved this study. All subjects provided their informed written consent for participation in the study.

**Protocol**

An experienced respiratory physician examined all the subjects. Thereafter, spirometry with bronchodilation test (Sensor Medic Vmax 22D, Sensor Medics Corporation, Yorba Linda, California, USA)[21] and skin prick tests to common aeroallergens ( Soluprick SQ®, ALK-Abelló, Hörsholm, Denmark) were performed. Atopy was defined as at least a 3 mm wheal reaction to any of the allergens.[22] The symptom questionnaire was completed under the guidance of a research nurse. During the same day, the hypertonic histamine challenge was performed. The subjects with asthma, suspicion of asthma, COPD, and rhinitis performed ambulatory PEF monitoring for two weeks, three times daily. The response to 0.2 mg salbutamol (Buventol Easyhaler®, Orion Ltd., Orion Pharma, Helsinki, Finland) was assessed every morning during the second week.[23]

**Hypertonic histamine challenge**

Three technically acceptable forced expiratory manoeuvres (model M9449; Medikro Ltd; Kuopio, Finland) were performed, and the largest FEV1 was used as the baseline value. A portable ultrasound nebulizer with a measured output of 0.44-0.48 ml/min (Omron U1; Omron LTD; Tokyo, Japan) was administered. The challenge was started with inhalation of hypertonic phosphate buffered saline aerosol for two minutes. After a 90 second follow-up period, two technically satisfactory forced expiratory manoeuvres were performed. In the subsequent inhalations, histamine diphosphate (Histamini phosphas; Ph.Eur., University Pharmacy; Helsinki, Finland) was dissolved in hypertonic phosphate buffered saline solution at doubling concentrations from 0.0075 to 4.0 mg/ml. The osmolality of the hypertonic histamine solutions was 1522-1577 mOsm/kg. The challenge was continued until the FEV_1_ had fallen ≥20% from the baseline value, or up to the inhalation of the final solution. The airway responsiveness to hypertonic histamine was expressed as the provocative concentration of histamine to induce a 20 % fall in FEV_1_ (PC_20_) which was estimated by linear interpolation. For statistical analyses, an arbitrary concentration of 8 mg/ml was used as the PC_20_ value in those subjects who did not respond to the final histamine concentration of 4 mg/ml. The coughs were manually recorded during the challenge by a research nurse. The session was video recorded to enable subsequent cough counting to determine the test reliability.

**Statistical analysis**

The cumulative number of coughs during the entire challenge was calculated. This value was divided by the achieved final histamine concentration to obtain the coughs/concentration ratio (CCR), this being expressed as coughs per mg/ml. Coughing frequency (Cf) was defined as the cumulative number of coughs divided by the cumulative duration of the observation period, and it was expressed as number of coughs per minute (maximum 38.5 min consisting of 12 nebulations). To exclude the possible cough-provoking effect of bronchoconstriction, we calculated the mean Cf at that stage of the challenge when the fall in FEV_1_ was < 5% from the baseline value (Cf_under5_).

Kolmogorov-Smirnov test was used for normality testing, and logarithmic transformations of cough variables and PC_20_ values were used in statistical analyses. The values are expressed as geometric mean (95% CI) if not otherwise stated. The differences in cough responses between groups were evaluated by using one-way ANOVA with Dunnett’s t-test and with Bonferroni correction when needed. Receiver operator characteristic (ROC) curves were constructed for all of the cough variables and univariate Z-score test was obtained utilizing the area under curve (AUC) values to determine the index with the best discriminative power. Into the latter analysis, asthmatic and symptomatic control group were included. Possible effects of confounding factors on utility of cough variables and on diagnostic value of CCR were evaluated by logistic regression analysis. Differences in cough frequency between groups were assessed with Chi square test. A p-value of <0.05 was considered to be statistically significant. All analyses were carried out using SPSS for Windows 14.0 (SPSS Inc. ™, Chicago, USA).

**RESULTS**

The coughs/concentration ratio (CCR) for asthmatics was 302(166-562) coughs per mg/ml, for the symptomatic controls 29.5(20.0-43.7) coughs per mg/ml and for the healthy subjects 6.61(3.02-14.5 coughs per mg/ml. The CCR of asthmatic and symptomatic groups differed statistically significantly from each other and from CCR of the healthy subjects (both p<0.001) (Figure 1.). The values of Cf_under5_ of asthmatics (17.8(11.5-26.9) coughs/min) were statistically significantly higher than those of the healthy subjects (6.31(3.47-11.5) coughs/min, p=0.016), but did not differ from those of the symptomatic individuals. CCR values of different symptomatic subgroups are expressed in table 2.

The univariate Z-score test showed that the AUC of CCR was 0.850, this being statistically significantly larger than those of the Cf and Cf_under5_ variables (data not shown). Therefore, further analysis of the results concentrated on CCR values. The ROC curve for the logCCR determined from the videoed coughs indicated that 1.84 (area under the curve 0.977, p<0.0001) represented the optimal cut-off point for logCCR to separate asthmatic subjects from healthy controls with sensitivity of 80% and specificity of 96%. Moreover, 2.22 would be the optimal cut-off point for logCCR to separate the asthmatic subjects from the symptomatic control group with a sensitivity of 70% and specificity of 86%. (Figure 2)

The logistic regression analysis was conducted between the asthmatic subjects and the symptomatic control group consisting of subjects with symptoms suggestive of asthma but without objective evidence of reversible airway obstruction in at least 6 months follow-up, and of subjects with COPD, parenchymal lung disease, persistent rhinitis, and chronic heart failure. The analysis confirmed that CCR independently had a good diagnostic accuracy in the differential diagnosis of asthma (p<0.001). It also revealed that smoking, gender, atopy and baseline FEV_1_ had no significant confounding effect on diagnostic utility of cough measurements.

**DISCUSSION**

As far as we are aware, this is the first study to evaluate the feasibility of using airway challenge induced cough in the differential diagnosis of asthma in a clinically relevant, unselected patient population. In agreement with our previous report [9], the CCR of the asthmatic subjects was statistically significantly higher than that of the healthy subjects. Furthermore, the CCR of the asthmatic subjects was also higher than that of the symptomatic subjects who had asthma-like symptoms due to other respiratory or cardiac disorders. These results suggest that hypertonic histamine challenge induced cough could be utilised in the differential diagnosis of asthma. In addition, the independence of challenge induced cough from patient’s effort and co-operation suggest that it has benefits over spirometry.

This study showed that CCR as a marker of cough independently exhibited a good prognostic value in the differential diagnosis of asthma. In agreement with our previous report, the cough response distinguished the asthmatic subjects from the healthy controls with a specificity of 96% and a sensitivity of 80%.[8] Moreover, if one relied only on cough measurements then the specificity and sensitivity to separate the asthmatics from the heterogeneous symptomatic control group were 86% and 70% respectively, which is well achieved in this clinically very demanding patient population. In our recent study utilising spirometrically assessed PC_20_, the sensitivity of the HHC was 81% and the specificity was 70% with the cut-off value 0.83 mg/ml in this same study population.[15] Methacholine, perhaps the most popular airway challenge in assessing airway hyperresponsiveness nowadays, has shown slightly better values for sensitivity (87.5-91%) and specificity (86.7-90%).[24,25] However, the patient material in metacholine studies may not have corresponded to the real life clinical situation as well as that of the present study where the symptomatic controls reported frequently as much cough as the asthmatic subjects, for example. In Finland most of the asthma diagnoses are set in primary care as this is the intention of the National Asthma Programme [26]; the reason why the patients are referred to a specialist in a tertiary referral centre is almost invariably due to diagnostic difficulties. Both direct [27] and indirect [2,28] inhalation challenge tests have been shown to have limited value in the differential diagnosis of mild and atypical asthma in Finnish out-patient clinic material. This is partly due to the high prevalence of mild to moderate airway hyperresponsiveness which is known to be associated with many common respiratory diseases [13,29-31] in the adult population. It is possible that examination of hypertonic histamine challenge induced cough may help to overcome some of the present diagnostic problems.

The present results emphasize the utility of CCR over other previously introduced cough variables in the differential diagnosis of respiratory diseases. [9] The CCR occuring in association with hypertonic histamine challenge consists of two components, the bronchoconstriction induced cough and the cough induced by the elevated osmolarity of the epithelial lining fluid. [32] The bronchoconstriction induced cough is a physiological response common to both healthy and asthmatic subjects. On the contrary, cough induced by hypertonicity is a pathological feature associated with asthma. The sensitivity to histamine is elevated in smooth muscle cells of asthmatics compared to those of the controls. This sensitivity leads to bronchial obstruction and stimulation of coughs at lower histamine concentrations in asthmatics. At the same time, the sensitivity of cough receptors to a hyperosmolar stimulus is elevated among asthmatic subjects but is not disturbed among controls with respiratory symptoms due to other causes. [9,10]

Hypertonic histamine challenge induced cough response is independent of the patient’s effort and co-operation. In line with report of Toelle et al., we have demonstrated that HHC is a safe and well tolerated challenge method. [33,34] This suggests that HHC induced cough could be utilised in diagnosing asthma in patients with poor ability to be subjected to spirometry which is often the case with small children and elderly subjects. However, in agreement with an earlier report evaluating children, we were not able to predict the magnitude of the challenge induced bronchoconstriction with the number of coughs alone (data not shown). [6]

To further improve the feasibility of using hypertonic histamine challenge, spirometry should be replaced by some other safety measurement. In previous attempts to simplify protocols for inhalation challenges, several non-invasive techniques requiring only passive co-operation have been utilised. For eaxmple, the pulse oximetry and chest auscultation are interesting, since they can be used in the inhalation challenge test conducted in primary care, even in field studies. The decrease of the arterial oxygen level and its association to changes in airflow measurements during methacholine challenge is also well documented [8]. Though the sensitivity of the pulse oxymetry is reported to be at least as good as that of the FEV_1_ in recognising bronchoconstriction, it is not recommended to be used as the only safety measurement. [5] Chest auscultation has been utilised successfully in detecting challenge induced wheezing as a challenge end-point in children. [6,35] However, a significant fall in FEV_1_ may occur without any wheezing, suggesting that the chest auscultation is also not sufficient when used alone. A recent study concerning the feasibility of using methacholine challenge in children, supported the combination of auscultation, follow up of coughing and measurement of oxygen saturation in assessing the challenge end-point.[36] This kind of combination might be a useful option also in the context of hypertonic histamine challenge, and deserves further evaluation.

In conclusion, the present study demonstrates that the cough response to hyperosmolar airway challenges can be utilised in the differential diagnosis of asthma. Since this response is independent of patient co-operation, it may be especially useful among subjects who cannot perform spirometry in a reliable manner.

**Acknowledgements**: Ms. Raija Tukiainen, RN, is cordially thanked for her contribution to this study. Liisa Hyvärinen, MD, and Jouko Kokkarinen, MD, are thanked for submitting patients to this study.

**Conflicts of Interests:** None of the authors has any conflict of interest related to the article or to the research described.

**Funding:** This project has been funded by Kuopio University Hospital. In addition, Dr. Purokivi was awarded a personal scholarship for manuscript preparation from the Finnish Lung Health Association.

**REFERENCES**

1. Deal EC, Jr., McFadden ER, Jr., Ingram RH, Jr., Jaeger JJ. Hyperpnea and heat flux: initial reaction sequence in exercise-induced asthma. J Appl Physiol 1979;46:476-483.

2. Koskela HO, Hyvarinen L, Brannan JD, Chan HK, Anderson SD. Responsiveness to three bronchial provocation tests in patients with asthma. Chest 2003;124:2171-2177.

3. Pezzoli L, Giardini G, Consonni S et al.. Quality of spirometric performance in older people. Age Ageing 2003;32:43-46.

4. Adinoff AD, Schlosberg RT, Strunk RC. Methacholine inhalation challenge in young children: results of testing and follow-up. Ann Allergy 1988;61:282-286.

5. Bisgaard H, Klug B. Lung function measurement in awake young children. Eur Respir J 1995;8:2067-2075.

6. Springer C, Godfrey S, Picard E et al.. Efficacy and safety of methacholine bronchial challenge performed by auscultation in young asthmatic children. Am J Respir Crit Care Med 2000;162:857-860.

7. Wang J, Mochizuki H, Muramatsu R, Arakawa H, Tokuyama K, Morikawa A. Evaluation of bronchial hyperresponsiveness by monitoring of transcutaneous oxygen tension and arterial oxygen saturation during methacholine challenge in asthmatic children. J Asthma 2006;43:145-149.

8. Cockcroft DW, Hurst TS, Marciniuk DD et al.. Routine pulse oximetry during methacholine challenges is unnecessary for safety. Chest 2000;118:1378-1381.

9. Koskela HO, Kontra KM, Purokivi MK, Randell JT. Interpretation of cough provoked by airway challenges. Chest 2005;128:3329-3335.

10. Koskela HO, Martens R, Brannan JD, Anderson SD, Leuppi J, Chan HK. Dissociation in the effect of nedocromil on mannitol-induced cough or bronchoconstriction in asthmatic subjects. Respirology 2005;10:442-448.

11. Remes J, Miettinen H, Reunanen A, Pyorala K. Validity of clinical diagnosis of heart failure in primary health care. Eur Heart J 1991;12:315-321.

12. Kornmann O, Beeh KM, Beier J, Geis UP, Ksoll M, Buhl R. Newly diagnosed chronic obstructive pulmonary disease. Clinical features and distribution of the novel stages of the Global Initiative for Obstructive Lung Disease. Respiration 2003;70:67-75.

13. Shorr AF, Torrington KG, Hnatiuk OW. Endobronchial involvement and airway hyperreactivity in patients with sarcoidosis. Chest 2001;120:881-886.

14. Palombini BC, Villanova CA, Araujo E et al.. A pathogenic triad in chronic cough: asthma, postnasal drip syndrome, and gastroesophageal reflux disease. Chest 1999;116:279-284.

15. Purokivi M, Koskela H, Koistinen T et al. Utility of hypertonic histamine challenge in distinguishing difficult-to-diagnose asthma. The Clinical Respiratory Journal 2007;1:91-98.

16. Global strategy for asthma management and prevention. In: National Institute of Health, Lung and Blood Institute. NHLBI/WHO Workshop report. Washington DC;revised 2006.

17. Global initiative for chronic obstructive lung disease. 2006.http://coldcopd.org.

18. King Jr T, Costabel U, Cordieer J-F. Idiopathic pulmonary fibosis: diagnosis and treatment International consensus statement. Am J Respir Crit Care Med 2000;161:646-664.

19. Bousquet J, Van Cauwenberge P, Khaltaev N. Allergic rhinitis and its impact on asthma. J Allergy Clin Immunol 2001;108:S147-334.

20. Manolio TA, Baughman KL, Rodeheffer R et al.. Prevalence and etiology of idiopathic dilated cardiomyopathy (summary of a National Heart, Lung, and Blood Institute workshop. Am J Cardiol 1992;69:1458-1466.

21. Pellegrino R, Decramer M, van Schayck CP et al.. Quality control of spirometry: a lesson from the BRONCUS trial. Eur Respir J 2005;26:1104-1109.

22. Dreborg S, Backman A, Basomba A, Bousquet J, Dieges P, Malling H-J. Skin tests used in type I allergy testing. Position paper. Allergy 1989;44:1-59.

23. Koskela HO, Hyvarinen L, Brannan JD, Chan HK, Anderson SD. Coughing during mannitol challenge is associated with asthma. Chest 2004;125:1985-1992.

24. Hunter CJ, Brightling CE, Woltmann G, Wardlaw AJ, Pavord ID. A comparison of the validity of different diagnostic tests in adults with asthma. Chest 2002;121:1051-1057.

25. Berkman N, Avital A, Breuer R, Bardach E, Springer C, Godfrey S. Exhaled nitric oxide in the diagnosis of asthma: comparison with bronchial provocation tests. Thorax 2005;60:383-388.

26. Haahtela T, Tuomisto LE, Pietinalho A et al.. A 10 year asthma programme in Finland: major change for the better. Thorax 2006;61:663-670.

27. Sovijarvi AR, Malmberg LP, Reinikainen K, Rytila P, Poppius H. A rapid dosimetric method with controlled tidal breahing for histamine challenge. Repeatability and distribution of bronchial reactivity in a clinical material. Chest 1993;104:164-170.

28. Koskela HO, Rasanen SH, Tukiainen HO. The diagnostic value of cold air hyperventilation in adults with suspected asthma. Respir Med 1997;91:470-478.

29. Xu X, Rijcken B, Schouten JP, Weiss ST. Airways responsiveness and development and remission of chronic respiratory symptoms in adults. Lancet 1997;350:1431-1434.

30. Witteman AM, Sjamsoedin DH, Jansen HM, van der Zee JS. Differences in nonspecific bronchial responsiveness between patients with asthma and patients with rhinitis are not explained by type and degree of inhalant allergy. Int Arch Allergy Immunol 1997;112:65-72.

31. Brunnee T, Graf K, Kastens B, Fleck E, Kunkel G. Bronchial hyperreactivity in patients with moderate pulmonary circulation overload. Chest 1993;103:1477-1481.

32. Higenbottam T. The ionic composition of airway surface liquid and coughing. Bull Eur Physiopathol Respir 1987;23 Suppl 10:25s-27s.

33. Toelle BG, Li J, Dalton M, Devadason SG. Subject discomfort associated with the histamine challenge in a population study. Respir Med 2002;96:990-992.

34. Koskela HO, Kontra KM, Purokivi MK, Randell JT. Hypertonicity of the challenge solution may increase the diagnostic accuracy of histamine challenge. Respir Med 2005;99:726-734.

35. Godfrey S, Uwyyed K, Springer C, Avital A. Is clinical wheezing reliable as the endpoint for bronchial challenges in preschool children? Pediatr Pulmonol 2004;37:193-200.

36. Kivastik J, Gibson AM, Primhak RA. Feasibility of shortened methacholine challenge in preschool children. Pediatr Pulmonol 2006;41:146-150.

37. Viljanen A, Halttunen P, Kreus K-E, Viljanen BC. Spirometric studies in non-smoking, healthy adults. Scand J Clin Lab Invest. 1982;42:5-20.

Table 1. Characteristics of the subjects.

|  | Healthy | Asthmatics | Symptomatic  controls |
| --- | --- | --- | --- |
| n | 25 | 30 | 82 |
| Gender  M/F | 12/13 | 6/24 | 39/43 |
| Age | 42  (22-59) | 48  (25-73) | 47  (19-74) |
| Smoking  current  previous | 10  1 | 7  12 | 14  24 |
| Pack-years | 12  (5-20) | 10  (0.3-40) | 16  (1.5-45) |
| Symptom duration  (months) |  | 48  (4-180) | 47  (0-150) |
| Cough during  the preceding 2 weeks | 4 (16%) | 25 (81%)^‡^ | 53 (65%)^‡^ |
| Atopy§ | 8 | 13 | 22 |
| FEV_1_ %# | 97  (77-116) | 89  (66-118) | 93  (65-122) |

n= number of subjects. Age, pack-year (=20 cigarettes per day for one year), FEV_1_ and symptom duration are expressed as mean (range). ‡ asthmatics versus symptomatic controls, p> 0.05 § Atopy is assessed as ≥ 3 mm mean wheel diameter in skin prick test to at least one common aeroallergen. # Pre-bronchodilation values are shown. Predicted values are those of Viljanen et al. [37]

Table 2. Coughs to concentration ratio to different diagnostic subgroups expressed as geometric mean with 95% confidence interval.

| **Group** | **n** | **geometric mean** | **95% CI** | **p** |
| --- | --- | --- | --- | --- |
| Asthma | 30 | 302 | 166-562 | p<0.001 |
| Suspicion of asthma | 26 | 34.7 | 19.1-61.7 | p=2.205 |
| COPD | 7 | 69.1 | 18.6-251 | p<0.001 |
| Parenchymal lung disease | 15 | 11.5 | 4.07-32.4 | p<0.001 |
| Rhinitis | 21 | 49.0 | 19.5-120 | p<0.001 |
| Cardiac failure | 13 | 24.0 | 8.71-64.6 | 0.021 |
| Healthy non-smokers | 15 | 6.31 | 1.78-22.4 | p<0.001 |
| Healthy smokers | 10 | 6.92 | 2.34-20.4 | p<0.001 |

n=number of the subjects. p<0.05 is considered statistically significant in comparison of asthmatic subjects to other separate subgroups.

Figure 1. The coughs/concentration ratio (the cumulative number of coughs during the entire challenge divided by the achieved final histamine concentration) of asthmatic subjects was statistically significantly higher than that of the healthy or the symptomatic controls (p<0.001, respectively). The horizontal lines express geometric means.

Figure 2. ROC curve of the coughs/concentration ratio. If one wishes to compare asthmatic subjects to symptomatic controls, 2.22 is the optimal cut-off point. The area under the curve is 0.850.
